# Detecting Hotspot Information Using Multi-Attribute Based Topic Model

**DOI:** 10.1371/journal.pone.0140539

**Published:** 2015-10-23

**Authors:** Jing Wang, Li Li, Feng Tan, Ying Zhu, Weisi Feng

**Affiliations:** School of Computer and Information Science, Southwest University, Chongqing, China; Tianjin University of Technology, CHINA

## Abstract

Microblogging as a kind of social network has become more and more important in our daily lives. Enormous amounts of information are produced and shared on a daily basis. Detecting hot topics in the mountains of information can help people get to the essential information more quickly. However, due to short and sparse features, a large number of meaningless tweets and other characteristics of microblogs, traditional topic detection methods are often ineffective in detecting hot topics. In this paper, we propose a new topic model named multi-attribute latent dirichlet allocation (MA-LDA), in which the time and hashtag attributes of microblogs are incorporated into LDA model. By introducing time attribute, MA-LDA model can decide whether a word should appear in hot topics or not. Meanwhile, compared with the traditional LDA model, applying hashtag attribute in MA-LDA model gives the core words an artificially high ranking in results meaning the expressiveness of outcomes can be improved. Empirical evaluations on real data sets demonstrate that our method is able to detect hot topics more accurately and efficiently compared with several baselines. Our method provides strong evidence of the importance of the temporal factor in extracting hot topics.

## Introduction

Microblogging, has quickly emerged as a popular and common form of social media in recent years because of its ease of use and convenience. With more and more people using this platform, it is increasingly important to extract hot topics from these short texts so that users can grasp interesting hot topics conveniently and quickly. However, detecting hot topics from these short texts poses new challenges. Unlike normal documents, these messages are usually noisier, less topic-focused and much shorter. Processing these texts efficiently is also a problem since large number of messages are posted by every minute. Moreover, fewer messages on microblogs are valuable for hot topic detection because massive messages range from coverage of serious political events and daily life such as weather, foods, emotions, and so on. Therefore, traditional topic analysis methods usually fail to achieve the desired accuracy.

Several studies attempted to solve the issues mentioned above. External knowledge, such as Wikipedia or news articles, is employed to expand and enrich the context of the short messages [1][2][3]. Their experiments show that an appropriate external knowledge can really enhance the performance. Some others develop to take advantage of the future of information spread in complex networks [4][5]. Prasad [6] has indicated that the attributes of microblogs, such as posted time, report time and repost count and comment count, always contain mass of useful information. Utilizing these attributes has shown positive improvement in the hot topic detection [7][8][9].

Topic models can express texts as a special distribution of hidden topics. So we can fully use the latent semantic of texts to reduce the limit caused by sparse data feature of the short text. However, traditional topic models can not model microblogs data very well without considering its inner affiliated information. It would be interesting to align the temporal dimension to the corresponding information (e.g., Tencent microblogs, Sina microblogs, Twitter). In this paper, we formalize the problem of hot topic extraction into a generative model, and propose a novel probabilistic generative model based on LDA [10], called MA-LDA. Our method incorporates the time and tag attributes into LDA. Usually, hot topics have a obvious and important feature that they always appear more frequently in a time interval. By using the time attribute, we can filter out the hot topic related words. Furthermore, utilizing hashtag attribute can enrich the expressiveness of the results based on LDA model.

In MA-LDA, topics are extracted from the messages only with a high predefined comment count and retweet count because hot topics related messages are always retweeted and commented with high frequencies.

The main contributions of this paper can be summarized as follows:

- We formally present a novel framework to extract hot topics. Firstly, we filter out messages with a predefined comment and retweet count. Then we use MA-LDA to extract hot topics.
- We propose an innovative topic model which can exploit time information of messages. Furthermore, an effective time distribution computing method is proposed.
- Experimental results show that our proposed framework can effectively address the challenging issues in the hot topics extraction problems in microblogs, significantly outperforming the two baselines mentioned in this paper.

## Related Work

Hot topic extraction is an important task for analyzing social media. In order to achieve satisfactory results, many classic models are utilized, such as single-pass clustering [11], latent semantic analysis (LSA) [12], latent dirichlet allocation (LDA) [10] and full-space clustering or subspace clustering algorithm [13]. Furthermore, as hot terms always appear more frequently in a time interval than others, the task of detecting topics in time-stamped text data has become the focus of recent studies. Up to now, there are mainly two methods of time-stamped text data analysis. One method first pre-divides the data into discrete time slices, and then fits a separate topic model in each slice such as the process in [14]. As further extensions, the work in [15] extends a time-series form of susceptible-infectious-recovered (SIR) model to monitor microblog emerging outbreaks, in which the SIR model has been widely applied in propagation in complex networks [16][17]. However, this method is of little use in identifying actual hot topics. Similar to the study of information evolution in [18][19], the other method firstly computes the temporal distribution of terms, then uses topic model to extract hot topics. Cataldi et al. [7] define the term as hot topic related if it frequently occurs in the specified time interval and it was relatively rare in the past. Additionally, they also evaluate the authority of a user by analyzing the social relationships in the network in order to get a more accuracy result. Chen et al. [20] compare the TF*PDF [21] of a word in different time slices to decide whether the term is hot or not. While, it is time consuming to compute TF*PDF of every term in every time interval, and is non-adaptive to the microblogging data. In this paper, we mainly focus on the latter idea.

Because of the shortness and informality in microblogs, directly applying conventional topic models (e.g. LDA and PLSA) often fails to achieve expected results. Many studies have shown that the model based on LDA can deal well with sparse and short data by discovering underlying topic structures in microblogs dataset such as [22][23]. To this end, many improvement measures have been adopted based on the standard LDA. Zhang et al. [9] take advantage of availability of LDA to expand the text feature space, and then use frequency statistics for topic ranking. They combine the time with retweet and comment count attributes to re-rank the key terms probability over topics based on the result of LDA. In addition, semantic analysis on microblogs is an essential improvement measure in efficient topic detection. In addition to the words’ co-occuring relations, the work in [24] also exploits sematic or syntax relation between words, which significantly enhances the dependence between their topics. By employing LDA to enrich and expand the feature space of Web segments semantically, Phan et al. [1] achieved significant quality enhancement. Another study direction is to use label or hashtag information as the weakly-supervised information. Ramage et al. [25] show that by training LDA on labeled messages, they get a better performance in author-topic modeling. While it is time consuming and subjective because the generation of the training set requires human participation. Ramage et al. [8] present a scalable implementation of a partially supervised learning model which utilize hashtags in microblogs. Similarly, the works in [26][27][28] also integrate the hashtags into LDA, and they all achieve a better representation of the content. Our objective is similar to theirs, but we focus on considering all related variables in an unsupervised model. In this paper, we propose a new method MA-LDA, a topic analysis model incorporates time and tag attributes in microblogs into LDA.

## Framework

**Fig 1:**
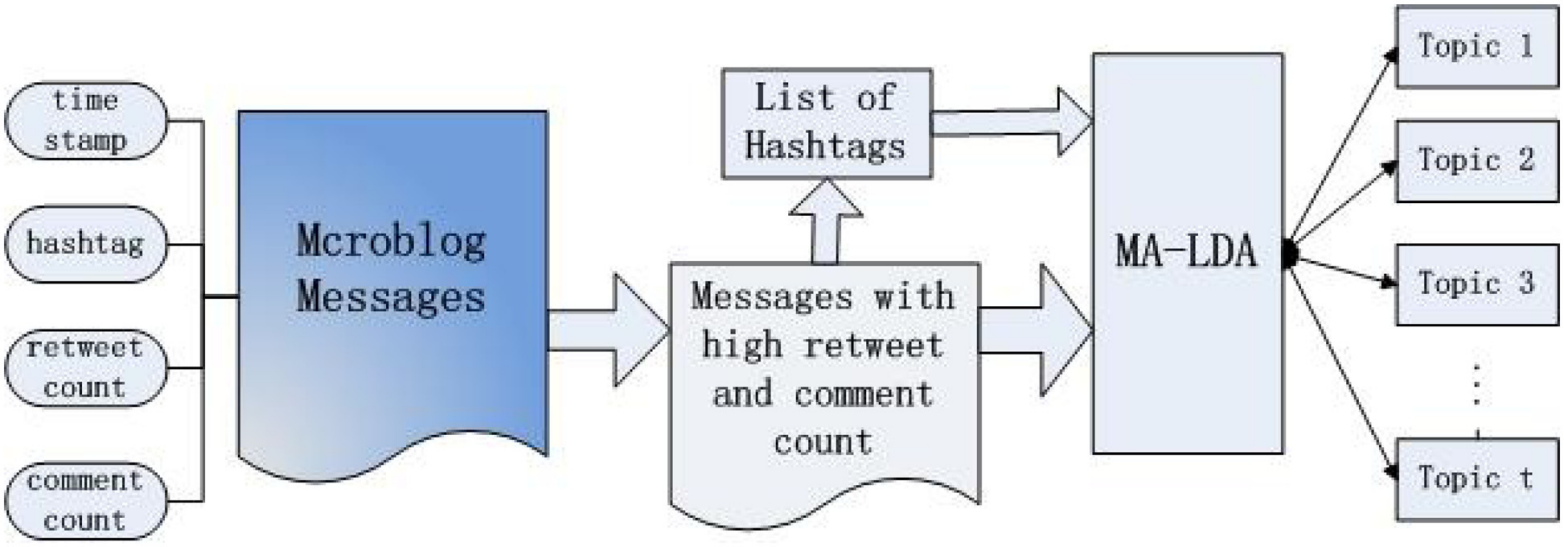
The framework of extraction hot topics from Microblog.

In this section, we present the framework that aims at identifying hot topics on microblogs more efficiently and accurately. The framework is depicted in Fig 1 which consists of following sub-components.

**Fig 2:**
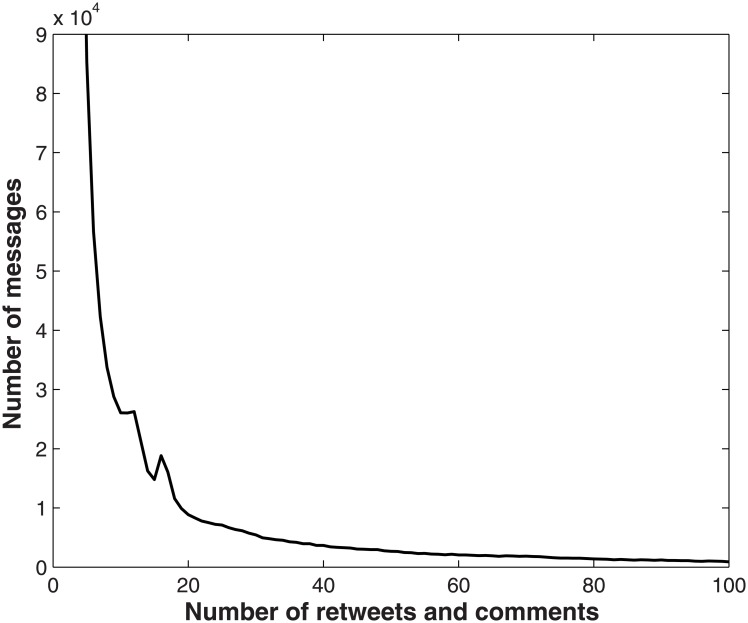
Distribution of the number of messages according to the number of retweet and comment.

Firstly, we filter out the messages from the raw dataset with a predefined retweet and comment count. Only the messages whose number of retweet and comment is larger than the predefined number are kept for hotspots detection. Generally, the number of retweet and comment for a certain message is a measure of the messages popularity. Moreover, hot topic related messages usually get a high retweet and comment count, but the daily life related messages can not get a high retweet and comment count in general. As a large number of messages is about users’ daily lives, filtering out these relatively useless information before doing topic analysis can greatly improve the efficiency of the process. Now the question is how to choose the potential retweet and comment count (which may affect the precision of hot topic detection) to contribute to the final results. Here we conduct an empirical study analysis. This study analyzes statistics on the number of messages according to the different number of retweet and comment. The data used in this study contains 11,285,538 messages. The statistics of the result are shown in Fig 2. From Fig 2, we can see that most of messages have a little number of retweet and comment and most of them get less than 20 retweets and comments. The total number of messages reduces exponentially while the threshold of the comment and retweet count is increasing. However, when the count is larger than 1000, the reduction of the total number of messages is not so obvious. According to the observation, we set the pre-defined count as 1000.

Though messages which contain hot topics are always retweeted and commented in a large number, the messages with high retweet and comment count are not always hot topic related. The next stage is performing topic analysis for these messages by MA-LDA. As Fig 1, the messages in microblogs have many useful attributes, such as time stamp, hashtag, retweet and comment count. Among these attributes, time is very important and valuable because hot topics tend to appear more frequently in one time interval than others. We incorporate time distribution into LDA so that the topic model can detect hot terms. With many noisy words, it is difficult to identify hot terms by time distribution. So designing a appropriate temporal distribution computing method is another problem which must be deal with.

Finally, we incorporate a list of hashtags into LDA model. The hashtags extracted from messages will be analyzed by MA-LDA. The topics produced by topic model consist of the n top-ranked words, rather than expressive sentences. Hashtags in microblogs may improve the expressiveness of outcomes, and also could be an indication of a topic that the message should stand for. Ramage et al. [25] introduce a topic model named Labeled LDA that constrains LDA by defining a one-to-one correspondence between LDAs latent topics and tags. The experiments show that Labeled LDA can achieve significant improvement. However, for the data used in their work, each document contains one or more tags. While in fact, not all messages in microblogs contain hashtags. Therefore, incorporating hashtags into LDA effectively is challenging.

## Multi-Attributes LDA

**Fig 3:**
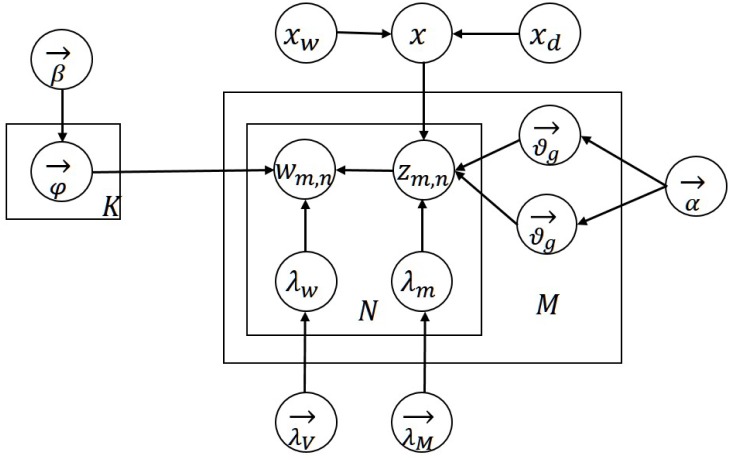
Graphical representation of MA-LDA model.

Topic models such as PLSA, LDA and their variants have been successfully applied in categorization [1], document & topic modeling [8], and opinion mining [29]. However, when dealing with microblogs, traditional topic models are unable to achieve satisfactory results. So we develop a multi-attribute topic model to consider multiple attributes and temporal information simultaneously. The graphical representation of the MA-LDA model is shown in Fig 3.

MA-LDA is a probabilistic graphical model based on standard LDA model. It describes a process for generating a time and hashtag based document collection. Likewise, MA-LDA models each document as a mixture of underlying topics and generates each word from one topic. Unlike LDA, there are two types of topics generated by MA-LDA. One is hot topics, and the other is general topics. The time related variable is computed to decide whether the generated word is from hot topics or general topics. Additionally, hashtags related parameters will affect the generation of both a certain topic and a certain word respectively from hot topic distribution over document and words distribution over hot topic. Table 1 lists the notations used in the model.

**Table 1:** Notations in MA-LDA.

*M*: the number of documents
*K*: the number of topics
*V*: vocabulary size
$\vec{\alpha },\vec{\beta }$: Dirichlet parameters
${\vec{\vartheta }}_{g},{\vec{\vartheta }}_{h}$: general,hot topic distribution over document *m*
$\Theta ={\left\{{\vec{\vartheta }}_{m}\right\}}_{m=1}^{M}$: a *M* × *K* matrix
${\vec{\varphi }}_{k}$: word distribution for topic *k*
$\Phi ={\left\{{\vec{\varphi }}_{k}\right\}}_{k=1}^{K}$: a *K* × *V* matrix
*N*_*m*_: the length of document *m*
*z*_*m*, *n*_: topic of nth word in document *m*
*ω*_*m*, *n*_:the *n*th word in document *m*
*x*: binary variable for time distribution
*x*_*w*_, *x*_*d*_: binary variable for time distribution about word and document
${\vec{\lambda }}_{M}$:vector of hashtags for documents
${\vec{\lambda }}_{V}$:vector of hashtags for words

### MA-LDA Algorithm

The generative process for MA-LDA is formally described in Algorithm 1. Getting *φ*_*k*_ for each topic k from a Dirichlet prior *β* remains the same as for traditional LDA. But when sampling topic distribution for each document, we use *x* to indicate whether the current word inherits topic from general topics *ϑ*_*g*_ (*x* = 0) or hot topics *ϑ*_*g*_ (*x* = 1). The value of *x* is affected by two binary variables *x*_*w*_ and *x*_*d*_. *x*_*w*_ is about the time distribution of a word. The process of estimating the value of *x*_*w*_ for *w*_*i*_ in vocabulary can be described as: Firstly, we random choose a set of messages from microblogs. Secondly, the time of the dataset is divided with *T* time slices, and we then count the value of *f*_*t*_ which is the frequency of occurrence of word *w* in (*t* + 1)^*th*^ time interval. Generally, a slice is defined as one day but can have other values. So time distribution for *w* is depicted as (*f*_*w*,0_, *f*_*w*,1_, ….., *f*_*w*, *t*_). Finally, the eigenvalue of time distribution for *w* is obtained as follows:

$$\begin{array}{c} S_{w}=\frac{\sum_{t}{\left(f_{w,t}-f_{avg}\right)}^{2}}{T\times f_{avg}^{2}} \end{array}$$ (1)

In Eq (1), *f*_*avg*_ is the average value of *f*_*w*, *t*_. The value of *S*_*w*_ for a hot term is likely larger than a general one. That is to say, we can extract hot terms from datasets by limiting the value of *x*_*w*_. With the experiments on real data, we observed that most hot terms can be identified when *S*_*w*_ > 0.5. So we define the value of *x*_*w*_ like this: *x*_*w*_ = 1 when *S*_*w*_ > 0.5, and *x*_*w*_ = 0 when *S*_*w*_ < = 0.5. Based on the value of *x*_*w*_, *x*_*d*_ can be decided as: If there are one or more words having *x*_*w*_ = 1 in document m, let *x*_*d*_ = 1. Otherwise, *x*_*d*_ = 0. This is dependent on the thought that words in document with hot terms are more likely hot words. Finally, we use logical operation “or” to combine *x*_*w*_ and *x*_*d*_, that is *x* = *x*_*w*_∣∣*x*_*d*_.

The hashtags in our model are associated with the sample of a certain hot topic and a certain hashtag related word. In this paper, we define two hashtag related vectors. One is the vector ${\vec{\lambda }}_{M}=\{\lambda_{1},\lambda_{2},...,\lambda_{m},...,\lambda_{M}\}$ for hashtags-documents, the value of element *λ*_*m*_ is either 0 or the occurrence count of the hashtag (which occurs in document *m*) on the overall dataset. The other is the vector ${\vec{\lambda }}_{V}=\{\lambda_{1},\lambda_{2},...,\lambda_{w},...,\lambda_{V}\}$ for hashtags-words. Likewise, the value *λ*_*w*_ in this vector is either 0 (when the current word *w* is not related to a hashtag) or a constant indicating the average number of occurrence count of all hashtags. Finally, all values in this two vectors will be drawn to control the hot topic sampling and word sampling together with the vector of ${\vec{\vartheta }}_{h}$ and ${\vec{\varphi }}_{h}$ respectively.

**Algorithm 1 Generative process for MA-LDA**

**Initialization**: time distribution value of *S*_*w*_, vector ${\vec{\lambda }}_{M}$ and ${\vec{\lambda }}_{V}$;

**for** each topic *k* ∈ 1, *K*: **do**

  sample mixture componentss ${\vec{\varphi }}_{k}∼Dir(\vec{\beta )}$;

**end for**

**for** each document *m*: **do**

  sample mixture proportion ${\vec{\vartheta }}_{g}∼Dir(\vec{\alpha })$;

  sample mixture proportion ${\vec{\vartheta }}_{h}∼Dir(\vec{\alpha })$;

  sample document length *N*_*m*_ ∼ *Poiss*(*ξ*);

**for** each word *n* ∈ [1, *N*_*m*_]: **do**

   compute *x* = *x*_*w*_∣∣*x*_*d*_ according to *S*_*w*_;

**if** *x* = 0 **then**

    sample topic index $z_{m,n}∼Mult({\vec{\vartheta }}_{g})$;

    sample term for word $\omega_{m,n}∼Mult({\vec{\varphi }}_{z_{m,n}})$;

**end if**

**if** *x* = 1 **then**

    sample topic index $z_{m,n}∼Mult({\vec{\vartheta }}_{h,\lambda_{m}})$;

    sample term for word $\omega_{m,n}∼Mult({\vec{\varphi }}_{z_{m,n},\lambda_{m,n}})$;

**end if**

**end for**

**end for**

As depicted in Fig 3 and Algorithm 1, especially for a document ${\vec{w}}_{m}$ (when *x*_*d*_ = 1), we can write the joint distribution of all known and hidden variables given the Dirichlet parameters as follows.

$$\begin{array}{c} p\left({\vec{w}}_{m},{\vec{z}}_{m},{\vec{\vartheta }}_{h,\lambda_{m}},\Phi |\vec{\alpha },\vec{\beta },{\vec{\lambda }}_{M},{\vec{\lambda }}_{V}\right)=p\left(\Phi |\vec{\beta },{\vec{\lambda }}_{V}\right)\prod_{n=1}^{N_{m}}p\left(w_{m,n}|{\vec{\varphi }}_{z_{m,n},\lambda_{m,n}}\right)p\left(z_{m,n}|{\vec{\vartheta }}_{h,\lambda_{m}}\right)p\left({\vec{\vartheta }}_{h,\lambda_{m}}|\vec{\alpha },{\vec{\lambda }}_{M}\right) \end{array}$$ (2)

Then the likelihood of the document ${\vec{w}}_{m}$ is obtained by integrating over ${\vec{\vartheta }}_{h,\lambda_{m}}$, Φ, and summing over ${\vec{z}}_{m}$ as follows.

$$\begin{array}{c} p\left({\vec{w}}_{m}|\vec{\alpha },\vec{\beta },{\vec{\lambda }}_{M},{\vec{\lambda }}_{V}\right)=\int \int p\left({\vec{\vartheta }}_{h,\lambda_{m}}|\vec{\alpha },{\vec{\lambda }}_{M}\right)p\left(\Phi |\vec{\beta },{\vec{\lambda }}_{V}\right)\cdot \prod_{n=1}^{N_{m}}p(w_{m,n}|{\vec{\vartheta }}_{h,\lambda_{m}},\Phi )d\Phi d{\vec{\vartheta }}_{h,\lambda_{m}} \end{array}$$ (3)

Moreover, the joint probability of the document ${\vec{w}}_{m}$ (when *x*_*d*_ = 0) is defined same as in LDA, which is defined as

$$\begin{array}{c} p\left({\vec{w}}_{m}|\vec{\alpha },\vec{\beta }\right)=\int \int p({\vec{\vartheta }}_{g}|\vec{\alpha })p\left(\Phi |\vec{\beta }\right).\prod_{n=1}^{N_{m}}p(w_{m,n}|{\vec{\vartheta }}_{g},\Phi ) \end{array}$$ (4)

### Estimation with Gibbs Sampling

The objective of training MA-LDA is to learn the proper latent variables $\vec{\vartheta }$ and $\vec{\phi }$ to maximize the likelihood of Eq (3) or Eq (4). In this paper, we take the widely used Gibbs Sampling technique [30]. Gibbs Sampling is a special case of Markov-chain Monte Carlo (MCMC) [31] and often yields relatively simple algorithms for approximate inference in high-dimensional models such as LDA. Gibbs sampling algorithm iterates through each word in the document corpus by staring with a random topic assignment $\vec{z}$. In each step, the algorithm samples a topic assignment for a word *w* conditioned on the topic assignments of all other words. Here, we only show the most important formula that is used for topic sampling with regard to a word. Specifically, for the word *t* in document m (when *x* = 1), the conditional probability of *z*_*t*_ = *h* is given by:

$$\begin{array}{c} p\left(z_{h,i}|x_{w}^{t}=1,z_{h,\neg i}\right)=\frac{n_{h,\neg i}^{\left(t\right)}+\beta }{\sum_{v=1}^{V}\left(n_{h,\neg i}^{\left(v\right)}+\beta \right)}\times \frac{n_{m,\neg i}^{\left(h\right)}+\alpha }{\sum_{j=1}^{H}\left(n_{m,\neg i}^{\left(j\right)}+\alpha \right)} \end{array}$$ (5)

where “¬” indicates excluding that instance from counting; $n_{h,\neg i}^{\left(t\right)}$ denotes the number of times the word t has been generated by hot topic *h*; $\sum_{v=1}^{V}(n_{h,\neg i}^{\left(v\right)})$ is the total number of words assigned to hot topic *h*; $n_{m,\neg i}^{\left(h\right)}$ is the number of words in document m assigned to hot topic *h*; $\sum_{j=1}^{H}(n_{m,\neg i}^{\left(j\right)})$ is the total number of words in document *m*; For the words in document *m* (when x = 0), the probability of $p(z_{g,i}|x_{w}^{i}=0,x_{d}^{i}=0,{\text{z}}_{g,\neg i})$ can be analogously defined as Eq (5). After finishing Gibbs Sampling, two matrices $\Theta =\{{\vec{\vartheta }}_{h,\lambda_{m}}\}$ and $\Phi =\{{\vec{\varphi }}_{h,\lambda_{w}}\}$ are computed as follows, while the computation of two general topic related matrices are consistent with standard LDA.

$$\begin{array}{c} {\varphi_{h,\lambda_{t}}}^{\left(t\right)}=\frac{n_{h}^{\left(t\right)}+\beta +\lambda_{t}}{\sum_{v=1}^{V}\left(n_{h}^{\left(v\right)}+\beta \right)+\lambda_{t}} \end{array}$$ (6)

$$\begin{array}{c} {\vartheta_{h,\lambda_{m}}}^{\left(m\right)}=\frac{n_{m}^{\left(k\right)}+\alpha +\lambda_{m}}{\sum_{j=1}^{K}\left(n_{m}^{\left(j\right)}+\alpha \right)+\lambda_{m}} \end{array}$$ (7)

Where the parameter *h* in Eq (7) is the specified hot topic *h* which maximize the $\varphi_{h,t}^{\left(tag\right)}$ in Eq (6) for the same document *m*. For our topic model or standard LDA, we usually use the n top-ranked words of a topic distribution for words to represent the final topic. So the usage of hashtag related parameter in Eq (6) can increase the probability of hashtag words in word-topic matrix. Meanwhile, this effect not only makes the outcome of MA-LDA easier to understand, but also benefits the result of hot topic detection.

## Experiments

In this section, we conduct several experiments to show that our MA-LDA model performs better than the two baselines on real-world datasets. One is proposed in [9] which we called TFLDA, and the other is standard LDA.

### Data Preparation

Two datasets Tencent microblogs (http://blog.qq.com/) and Twitter (https://twitter.com/) are used in our experiments. The Tencent Microblogs are crawled from several different “micro-channels” (http://t.qq.com/) on Tencent microblogs platform. It contains 11,285,538 messages which were posted by users from July 2 to July 14 in 2013. The second dataset is a subset of Twitter corpus in [23] which contains 3,844,612 messages.

In order to effectively detect hot topics from microblogs, we first draw messages from the raw data with a pre-defined threshold of retweet and comment count. In total, 400,000 messages are chosen randomly to compute time distribution of words. Specifically, for the process of word segmentation needed in Chinese microblogs, the ICTCLAS (http://www.ictclas.org/) is used for this paper. After extracting hashtags, namely the vocabulary between two “#” marks (Tencent microblogs) or following with one “#” mark (Twitter), other terms like the punctuation marks, stop words, links and other non-words in the original microblog datasets are removed in data preparation. The hashtag words are part of contents of the original messages in our method.

**Fig 4:**
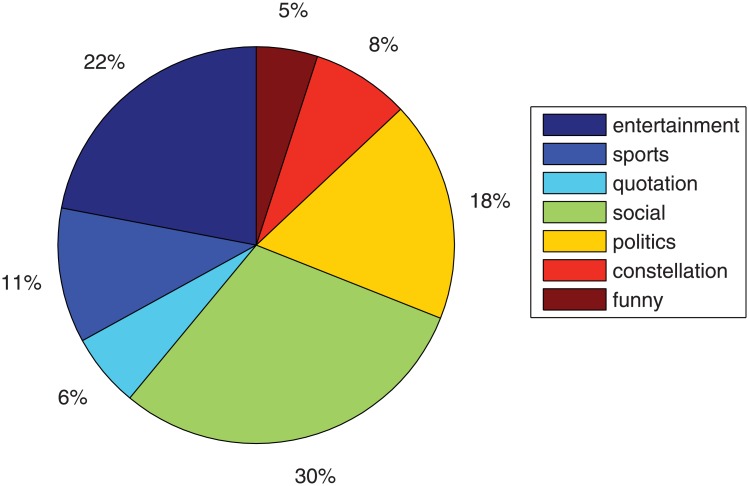
Category distribution of messages.

Table 2 summarizes the statistic information of two datasets in the experiment. Intuitively, the Twitter data contains more documents. The Tencent data are divided into seven categories according to their respective “micro-channel”. Different categorizations and their proportion distribution are shown in Fig 4.

**Table 2:** Illustration of datasets.

Dataset	♯ docs	♯ unique words	♯ hashtags
Tencent	6,884	19,335	1,735
Twitter	100,000	212,142	21,277

### Experimental results and analysis

Similar to the evaluation methods on topic model in [32] and [33], MA-LDA is compared with the baselines in three aspects: document modeling, document classification, and hot topic detection. To verify the effectiveness of using the attribute of hashtags, we test the performance of MA-LDA with these settings: without hashtags (called MA-LDA1), with random hashtags (called MA-LDA2), with hotspots related hashtags only (called MA-LDA3) and with all hashtags (MA-LDA4). The parameter settings for the whole experiments in this paper are consistent. First, following with the settings in [1][34], the parameter *α* = 0.5 and *β* = 0.1. Then, due to the reason that our algorithm converge at 800th iteration, all these models were run for 800 iterations and results of last step were used for evaluation. More specially, because the method in TFLDA is based on standard LDA, the performance of document modeling and document classification on standard LDA and TFLDA are the same.

#### Document Modeling

Following the practice of most existing studies on topic model [10], our model and baselines are tested in document modeling, and evaluated by using perplexity. As described in [33], the objective of document modeling is generating a density estimation that describes the underlying structure of data. Perplexity is a canonical measure of goodness. In language modeling, it is used to measure the likelihood of the held-out test data to be generated from the underlying distribution of the model [30]. Lower perplexity value represents a higher likelihood. Formally, for a test set of M document, the perplexity is defined as:

$$\begin{array}{c} perplexity\left(D_{test}\right)=\exp\{-\frac{\sum_{d}\sum_{w\in d}\ln p(w)}{\sum_{d}\left|d\right|}\} \end{array}$$ (8)

Where *D*_*test*_ is the collection of the test dataset, and ∣*d*∣ is the length of document d. The probability of each word is computed as *p*(*w*) = ∑_*k*_ *p*(*k*, *w*) = ∑_*k*_ *p*(*k*)*p*(*w*∣*k*).

In our experiments, we held out one third of messages for test purposes and trained the models on the remaining two thirds of messages for all datasets. Because of different data size between two datasets, it is not proper to assume that they contain the same number of topics. Therefore, we test all three models on Tencent with topic number K = 10,15,20,25,30. For the other dataset, we test all three models with topic number K = 40,50,60,70,80.

**Fig 5:**
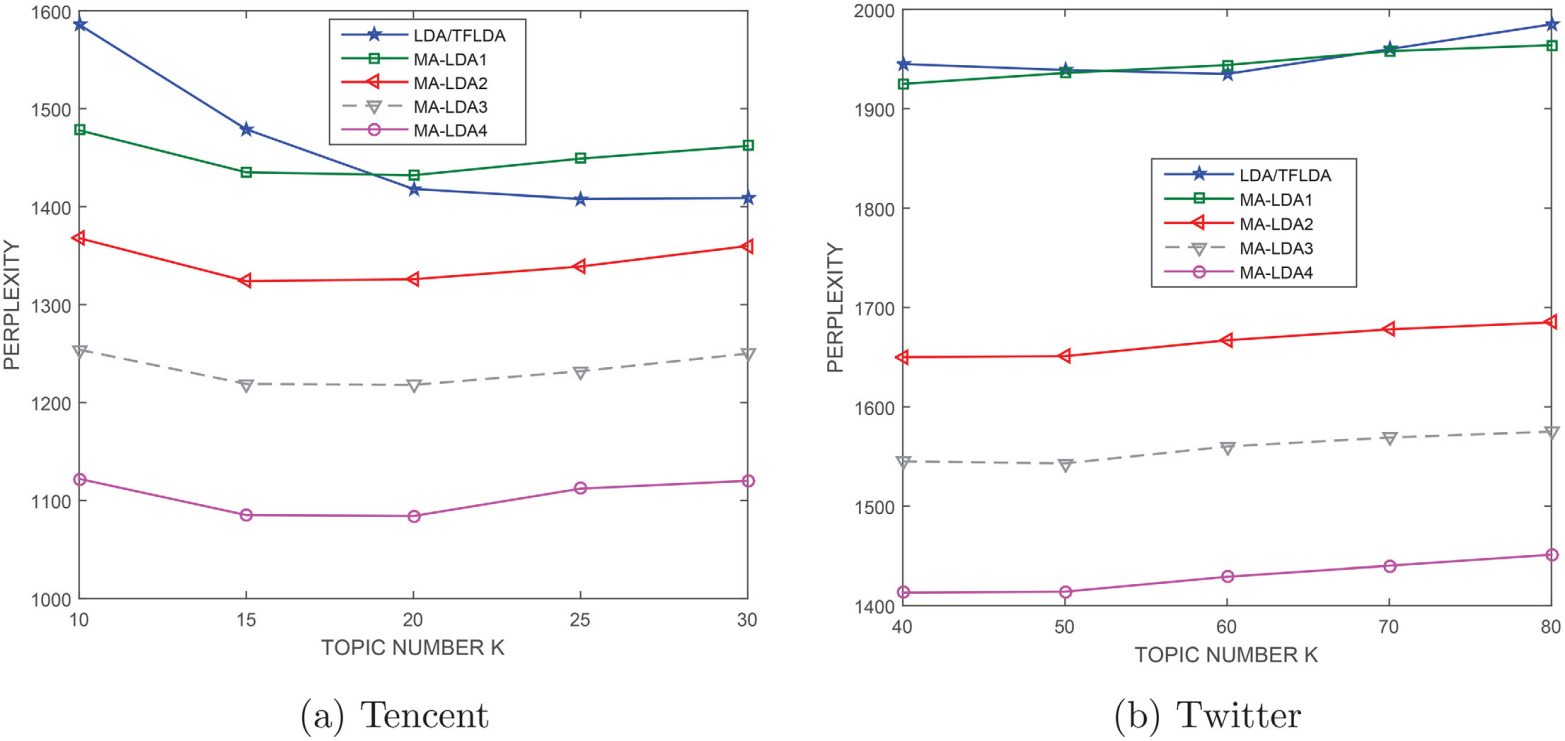
Experimental results on Tencent and Twitter data.

Fig 5(a) and 5(b) illustrate the perplexity of the models tested on the Tencent and Twitter, respectively. We repeat each experiment ten times with same settings, and the perplexity values are average values. Obviously, for all datasets, MA-LDA performs better than LDA and TFLDA regardless of what the setting of hashtags is. Besides, with the change of the number of topics, the change of perplexity values of MA-LDA is more stable compared with LDA. This means that our model is not sensitive to the change of topic number. Observing the different settings of hashtags in MA-LDA, we find that the performance of MA-LDA with hashtags (MA-LDA2, MA-LDA3, MA-LDA4) obviously outperforms that without hashtags (MA-LDA1). Meanwhile, both MA-LDA with all hashtags (MA-LDA4) and MA-LDA with hotspots related hashtags (MA-LDA3) outperform MA-LDA with random hashtags (MA-LDA2). One possible explanation is that MA-LDA3 and MA-LDA4 include more hashtags related words, random hashtags used in MA-LDA2, on the other hand, most likely result in irrelevant categories of hashtags. As a result, the former two produce higher perplexity value. From the comparison between the experimental results of Tencent and Twitter, we find that although the average value of perplexity on Tencent is lower than that of Twitter, MA-LDA produces much lower perplexity than LDA even without hashtags on Twitter data. In fact, the improvement of MA-LDA on Twitter is greater than that of Tencent. The most likely reason is that Twitter data cover more information than Tencent. More importantly, higher accuracy of word segmentation on English words is most likely to lead to higher accuracy of topic distribution.

#### Document Classification

Except estimating the probability of unseen held-out document given some training documents, topic models are also typically evaluated by measuring performance on some secondary task, such as document classification or information retrieval [32]. Besides, the topic distribution over a document can be considered as a simplified description of the document in a new space spanned by the small set of latent variables [10]. Inspired by the above works, we test the performance of document classification on these topic models. For evaluation, classification accuracy and F1 measure are common measures [33]. According to the category distribution of Tencent messages in Fig 4, we conduct a seven class-classification problem on the Tencent data. Following the classification method in [1], we integrate the topic distribution *ϑ*_*m*_ = {*ϑ*_*m*,1_, *ϑ*_*m*,2_,…, *ϑ*_*m*, *K*_} and the original document *w*_*m*_ = {*w*_*m*,1_, *w*_*m*,2_,…, *w*_*m*, *N*_*m*__}, and train the MaxEnt classifier on the integrated data (following with their actual class label added by manually) by using JMaxEnt4 (http://jtextpro.sourceforge.net). MaxEnt was run five times using different 60–40% partitions of train-test sets. Five MaxEnt classifiers were trained using the same parameter setting and built on the training data according to different number of topics. The setting of topic number is same as in document modeling. In this experiment, there is another baseline which uses MaxEnt classifier trained on Tencent data without hidden topics. All of methods with hidden topics as well as the baseline classifier were evaluated on the test data.

**Fig 6:**
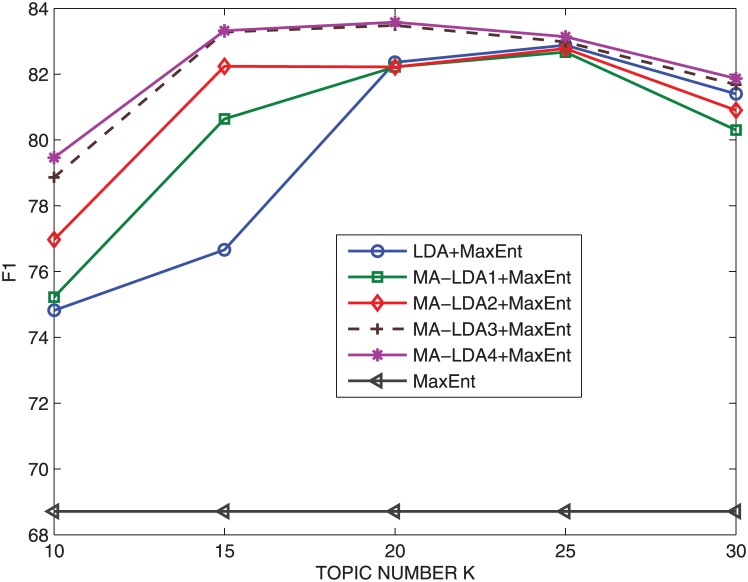
F1 changing with different topic numbers on Tencent.

The average F1 with different topic numbers for all models are given in Fig 6, and the performance in terms of classification accuracy over five different topic numbers are shown in Table 3. Fig 6 clearly shows that both LDA/TFLDA and MA-LDA perform significantly better than the baseline which only use word features. Overall, MA-LDA did slightly better than LDA. One possible reason is that MA-LDA has pre-classified the messages according to the x value of each document. By observing the performance among different settings of MA-LDA models, we can see that the result of MA-LDA3 approximates MALDA4, and these two methods have a slight improvement compared to MA-LDA2. The change of F1 measure with the increase of topic numbers is also shown in Fig 6. For all these topic models, the F1 measure increases gradually with 10, 15 topics, then remains stable between 15 and 25 topics, after that, it reduces gradually. In addition, MA-LDA outperforms LDA/TFLDA obviously when the topic number is 10 or 15, but it is slightly superior to LDA. This is because LDA produces more optimal result of topic distribution when the number of topic *K* is set to 20 or 25, respectively. We realize that the number of topic *K*, when set to 15 or 20, is the better setting for MA-LDA. Table 3 shows the evaluation metrics such as average, minimum, maximum, and standard error (SE) of the classification accuracy over different topic numbers on Tencent data. The standard error is defined as the standard deviation of the sampling distribution of the test data, and SE values of each corresponding method in Table 3 are computed according to their classification accuracy on five different topic numbers. The result mainly indicates that the classification accuracy on MA-LDA is quite stable with respect to the number of topics.

**Table 3:** The average,minimum,maximum,and standard error (SE) of the classification accuary over all topic numbers.

Method	accuracy	max accuracy	min accuracy	SE
LDA + MaxEnt	79.62	82.89	74.81	3.64
MA-LDA1 + MaxEnt	80.21	82.67	75.22	2.96
MA-LDA2 + MaxEnt	81.02	82.78	76.98	2.36
MA-LDA3 + MaxEnt	82.05	83.48	78.87	1.92
MA-LDA4 + MaxEnt	82.27	**83.61**	79.51	**1.70**

#### Hot Topic Detection

The objective of this set of experiment is to test the ability of our method to detect hot topics at the time of their arrival. We use one criterion to evaluate the performance of hot topic detection. This metric is Coverage Rate (CR) [20] which indicates how many hot topics are extracted for a specified period. The higher value of CR means the better performance. To study the effect of N top-ranked terms on the performance of hot topic detection, we choose top 10, 20, 30, 40 keywords for each topic to represent the final topics respectively. The CR is defined as:

$$\begin{array}{c} CoverageRate\left(CR\right)=\frac{ExtractedHotTopics}{ActualHotTopics}\times 100\% \end{array}$$ (9)

To assess the CR, we first manually extract genuine hot topics and their related keywords from the datasets according to the statistics of media recordation and hotspots rankings in microblogs platform in a specified period. Based on the optimal settings of topic numbers in the previous experiments, we set the topic numbers to be 20 on Tencent data and 50 on Twitter data. The results of CR on Tencent data and Twitter data is as Tables 4 and 5, respectively.

**Table 4:** CR results on Tencent.

Model	top10	top20	top30	top40
LDA	0.25	0.38	0.42	0.66
MA-LDA1	0.66	0.75	0.75	0.78
MA-LDA2	0.66	0.75	0.83	0.83
MA-LDA3	0.75	0.83	**0.91**	**0.91**
MA-LDA4	0.75	0.83	**0.91**	**0.91**
TFLDA	0.58	0.66	0.66	0.66

**Table 5:** CR results on Twitter.

Model	top10	top20	top30	top40
LDA	0.3	0.45	0.54	0.64
MA-LDA1	0.64	0.7	0.81	0.81
MA-LDA2	0.64	0.75	0.84	0.84
MA-LDA3	0.75	0.87	**0.92**	**0.92**
MA-LDA4	0.75	0.83	**0.92**	**0.92**
TFLDA	0.62	0.75	0.75	0.75

Tables 4 and 5 show that the more top words we retain, the higher CR we can achieve for LDA and MA-LDA, respectively. The reason behind is by including more factual hot topics related terms, we are able to extract more top-ranked terms from words-topic distribution. However, this experiment also shows that it is unnecessary to extract a large number of terms to cover the majority of hot topics because the CR hardly changes when the number of terms exceeded 30. In particular, the results from MA-LDA3 and MA-LDA4 show that the CR already exceeded 90 percent for genuine hot topics with 30 top-ranked terms. The top N keywords of a particular topic for the TFLDA is either semantically highly correlated or not related to the genuine hot topics. It is clear that the TFLDA is not sensitive to the number of top-ranked terms when it is larger than 20. The reason that a small number of hot topics makes little contribution to the final result is because there are not only few messages are included and related to them in corpus. From the experimental results of the detected hot topics, we can conclude that entertainments, sports, politics and social news categories are the most popular ones.

Compared with the standard LDA and TFLDA, our method produces the highest CR. Firstly, as described in the literature, the TFLDA is the result of a rearrangement of keywords (from the words distribution of topics) in accordance with word frequency statistics after first running LDA, thus producing more expressive and accurate results compared with LDA. We can observe that the TFLDA outperforms standard LDA by as much as 48.8%. However, based on the same reason, some detected topics in TFLDA are what users often talk about but are irrelevant to hot topics. Our model outperforms TFLDA by 21% on average. Both MA-LDA4 (with all hashtags) and MA-LDA3 (with hot hashtags) are optimum strategies in this paper.

At last, to intuitively show the results achieved through MA-LDA, some visualized results are listed in Table 6. A hot topic about the event of “Crash of Asiana Airlines Boeing 777” and its top-6 keywords from Tecent are selected as example. For convenience, we show them in English. This event is a significant aviation accident occurred on July 7, 2013 in San Francisco. The hashtags related keywords about this event include such as “Asiana Airlines”, “Boeing 777”, “air crash”, “crash of Asiana Airlines Boeing 777” and so on. The results in Table 6 indicate some important facts. Firstly, even with top-6 keywords, all settings of MA-LDA (with or without hashtags) can extract this hot topic successfully while it is not found in the results of standard LDA. Secondly, compared to MA-LDA without hashtags (MA-LDA1), the results produced by MA-LDA with hashtags (MA-LDA2, MA-LDA3, MA-LDA4) are more easily understood. In other words, the hashtag information can be used effectively to improve the expressiveness of a topic. Finally, MA-LDA3 and MA-LDA4 produce more hashtag related keywords with high probability value. This also proves that MA-LDA3 and MA-LDA4 are optimum strategies.

**Table 6:** Topic results about “Crash of Asiana Airlines Boeing 777” achieved by MA-LDA.

MA-LDA1	MA-LDA2	MA-LDA3	MA-LDA4
San Francisco	Asinana Airlines	Asinana Airlines	Asinana Airlines
Snow	air crash	Boeing 777	Boeing 777
airliner	San Francisco	aviation	airline
wrack	crash	air crash	air crash
naval vessel	camera	San Francisco	Ye Mengyuan
white paper	airliner	crash of Asiana Airlines Boeing 777	San Francisco

## Conclusion

In this paper, we introduce a new method called MA-LDA for hot topic detection on the platform of microblogs. Our model is based on LDA, and some attributions such as time stamps, hashtags as well as retweet and comment counts are also taken into account. We test the performance of our model on document modeling, document classification and hot topic detection, respectively. Standard LDA and the other baseline TFLDA mentioned in this paper are selected to compare with our model. In addition, there are four ways of settings on MA-LDA according to the usage situation of hashtags.

The experimental results on two datasets show that by incorporating time distribution and hashtags into LDA, our method performs better with greater stability than the standard LDA on both document modeling and document classification. More importantly, more hot topics are identified by our model. The obtained outcomes are more representative. It is clear that MA-LDA with all hashtags or hot hashtags is able to achieve better results.

In the future work, hot terms detection automatically based on the time distribution will be considered. Semantic relations between terms will be taken into consideration as well. Furthermore, we may consider the influence of dynamics or evolution of networks.
